# Potential RNA-dependent RNA polymerase inhibitors as prospective therapeutics against SARS-CoV-2

**DOI:** 10.1099/jmm.0.001203

**Published:** 2020-05-29

**Authors:** Rudramani Pokhrel, Prem Chapagain, Jessica Siltberg-Liberles

**Affiliations:** ^1^​ Department of Physics, Florida International University, 11200 SW 8th St, Miami, FL 33199, USA; ^2^​ Biomolecular Sciences Institute, Florida International University, 11200 SW 8th St, Miami, FL 33199, USA; ^3^​ Department of Biological Sciences, Florida International University, 11200 SW 8th St, Miami, FL 33199, USA

**Keywords:** sequence analysis, protein evolution, broadly neutralizing antivirals, docking

## Abstract

**Introduction.:**

The emergence of SARS-CoV-2 has taken humanity off guard. Following an outbreak of SARS-CoV in 2002, and MERS-CoV about 10 years later, SARS-CoV-2 is the third coronavirus in less than 20 years to cross the species barrier and start spreading by human-to-human transmission. It is the most infectious of the three, currently causing the COVID-19 pandemic. No treatment has been approved for COVID-19. We previously proposed targets that can serve as binding sites for antiviral drugs for multiple coronaviruses, and here we set out to find current drugs that can be repurposed as COVID-19 therapeutics.

**Aim.:**

To identify drugs against COVID-19, we performed an *in silico* virtual screen with the US Food and Drug Administration (FDA)-approved drugs targeting the RNA-dependent RNA polymerase (RdRP), a critical enzyme for coronavirus replication.

**Methodology.:**

Initially, no RdRP structure of SARS-CoV-2 was available. We performed basic sequence and structural analysis to determine if RdRP from SARS-CoV was a suitable replacement. We performed molecular dynamics simulations to generate multiple starting conformations that were used for the *in silico* virtual screen. During this work, a structure of RdRP from SARS-CoV-2 became available and was also included in the *in silico* virtual screen.

**Results.:**

The virtual screen identified several drugs predicted to bind in the conserved RNA tunnel of RdRP, where many of the proposed targets were located. Among these candidates, quinupristin is particularly interesting because it is expected to bind across the RNA tunnel, blocking access from both sides and suggesting that it has the potential to arrest viral replication by preventing viral RNA synthesis. Quinupristin is an antibiotic that has been in clinical use for two decades and is known to cause relatively minor side effects.

**Conclusion.:**

Quinupristin represents a potential anti-SARS-CoV-2 therapeutic. At present, we have no evidence that this drug is effective against SARS-CoV-2 but expect that the biomedical community will expeditiously follow up on our *in silico* findings.

## Introduction

Humanity is facing a global pandemic caused by a previously unknown coronavirus that recently crossed the species barrier [1] and is spreading by human-to-human transmission [2]. This novel coronavirus, SARS-CoV-2 (also known as 2019-nCoV), is a close relative of SARS-CoV [3], which caused an outbreak in 2002–2003 [4]. SARS-CoV had a high mortality rate of 10 %, but was limited to about 8000 cases in total [4]. MERS-CoV, another coronavirus that started an outbreak in 2012, has so far resulted in >2500 cases with an even higher mortality rate of ~35 % [5]. SARS-CoV-2, the cause of the ongoing COVID-19 pandemic, has proved to be the most infectious of these coronaviruses. As of 15 April 2020, it has caused over 2 million known infections and >130 000 deaths worldwide [6]. No vaccine or antiviral treatment for COVID-19 exists.

The positive-strand RNA genome of a coronavirus such as SARS-CoV and MERS-CoV encodes ~25 different protein products [7]. Four of these proteins have a structural function and assemble to form the capsid that encapsulates the viral genome. Upon infection, the viral RNA genome is released into a host cell, and by using the host’s translational machinery, the viral proteins are expressed. Several of these non-structural proteins (NSPs) form the replicase–transcriptase complex that works to replicate the RNA genome. Key proteins in the replicase–transcriptase complex are the RNA-dependent RNA polymerase (RdRP), the helicase and the exonuclease [8]. New copies of the viral genome and more structural proteins pack into new virions that are eventually released from the cell, on task to infect more cells. If the key proteins in the replicase–transcriptase complex can be prevented from replicating the RNA genome, new virions will not form. Recently we identified regions in the key proteins in MERS-CoV and SARS-CoV that could serve as targets for antiviral drugs [7]. We took an evolutionary approach to identify regions of at least five consecutive sites not just conserved in sequence but also in structural properties with low conformational flexibility to reduce the risk of a target being displayed for only a fraction of the time [7]. These targets represent potential drug-binding sites located in protein regions crucial for viral fitness and, therefore, conserved on long evolutionary time scales. Our study identified two to four such targets in the protease (NSP5), replicase (NSP7), helicase (NSP13) and exonuclease (NSP14), with nine targets identified in the RdRP (NSP12) [7].

In this work, we included SARS-CoV-2 in our analysis. We generated a conformational ensemble based on the 3D structure of the RdRP from SARS-CoV (PDB ID: 6NUR [9]), which is closely related and highly similar to the RdRP from SARS-CoV-2. The conformational ensemble was used for a virtual screen of the US Food and Drug Administration (FDA)-approved drugs against the conserved targets in RdRP. As the RdRP structure of SARS-CoV-2 just became available, we performed a second round of virtual screens with the FDA-approved drugs against the recently resolved RdRP conformation (PDB ID: 6M71 [10]). Altogether, these different screens revealed several drugs that are expected to inhibit RdRP catalytic activity. These drugs can, therefore, be repurposed as anti-COVID-19 therapeutics. Among the drug candidates, quinupristin, an intravenous antibiotic that has been in clinical practice against severe bacterial infections for a couple of decades and has a rather limited scope of side effects [11], appears to be particularly promising. Quinupristin is predicted to bind across the RNA tunnel of RdRP, suggesting that it may be able to inhibit viral RNA synthesis and thus block SARS-CoV-2 replication.

## Methods

### Phylogenetic analysis

A multiple sequence alignment of RdRP from SARS-CoV-2 (accession number: YP_009724389.1, range 4398–5324) and the RdRP sequences previously used to determine the target motifs [7] was constructed using mafft with L+INS+ i in Jalview [12]. The multiple sequence alignment was used to build a phylogenetic tree using PhyML with 100 bootstraps [13]. The best model of evolution for the dataset was determined by smart model selection [14]. The resulting phylogeny was rooted at mid-point.

### SARS-CoV-2 conservation

To determine the level of conservation of the proteins with target motifs, blastp searches with the regions from the SARS-CoV orf1ab polyprotein that represent NSP5 (protease), NSP7 (replicase), NSP12 (RdRP), NSP13 (helicase) and NSP14 (exonuclease) from [7] against SARS-CoV-2 in the nr database were performed, resulting in five sequence datasets. Each dataset was aligned using muscle [15] at EBI [16], resulting in a multiple sequence alignment (MSA) and its corresponding percentage identity matrix. The percentage identity matrices showed the overall conservation across all SARS-CoV-2 sequences in each dataset. The conservation of each target motif was extracted from the MSAs through visualization in Jalview [12].

### Target surface accessibility

The surface accessibility of the target motifs was determined using PyMol v. 1.8.4 [17] with the PyMol script *FindSurfaceResidues.py* [18] (default settings) from existing protein structures of SARS-CoV. The structures with PDB IDs 1UJ1 [19], 6NUR [9], 6JYT [20] and 5C8S [21] were used to represent NSP5 (protease), NSP7 (replicase) and NSP12 (RdRP), NSP13 (helicase) and NSP14 (exonuclease), respectively.

### Protein sequence and structure analysis

A blastp search with the *SEQRES* part of RdRP from SARS-CoV in PDB ID 6NUR [9] was performed against SARS-CoV-2 in the Refseq_protein database to determine the sequence similarity between SARS-CoV-2 and the actual part of RdRP from SARS-CoV that was included in the PDB structure. The differing sites were visualized in their structural context based on PDB ID 6NUR.

### Virtual screening of FDA approved drugs

#### Molecular dynamics simulations and preparation of protein conformations

The cryo-EM structure of SARS-CoV NSP12 (RdRP) bound to the NSP7 and NSP8 co-factors was taken from the Protein Data Bank (PDB ID: 6NUR) [9]. To incorporate the inherent flexibility, protein conformations were generated with all-atom molecular dynamics (MD) simulation. The system was prepared using the CHARMM-GUI web interface [22]. Standard MD simulation procedure was followed [23]. The protein was solvated with TIP3 water in a cubic box and neutralized with the NaCl ions added at a concentration of 0.15 mg l^−1^. The solvated system (protein, water and ions) contained ~276 000 atoms. All-atom MD simulations were performed with the CHARMM36m force field [24] using NAMD 2.13 [25]. The particle mesh Ewald (PME) [26] method was used to calculate the long-range ionic interactions. The covalent bonds involving hydrogen atoms were constrained by SHAKE [27]. A 10 000-step minimization and a 100 ps equilibration run were performed using a 1 fs time step. An NPT (constant pressure/temperature) production run was performed for 100 ns with a 2 fs time step at 300 K temperature and 1 atm pressure. A Nose–Hoover Langevin piston (with 50 fs period and 25 fs decay) was used for pressure control and the Langevin temperature coupling (with 1 ps^−1^ friction coefficient) was used for temperature control. Visualization and the PDB frame extraction from the trajectory were performed with VMD [28]. To prepare the receptor structures for virtual screening, 200 protein conformations were extracted from a 100 ns MD simulation. The PDB files were converted to pdbqt format using AutoDockTools 4.2 [29].

#### Preparation of drug files and virtual screening

The library of FDA-approved drugs was obtained from the e-Drugs3d web interface from Chemoinformatic Tools and Databases [30]. This database contains 1930 FDA-approved compounds in SDF format. Open Babel [31] was used to convert the SDF structures to pdbqt format with added polar hydrogen atoms. For virtual screening on the ensemble of SARS-CoV RdRP conformations, a docking configuration box was made by enclosing the RNA-binding tunnel region containing the evolutionarily conserved targets. Since all PDB conformations were aligned, the same configuration box could be used for docking in batch. Separately, all 1930 drugs were screened against the SARS-CoV-2 structure of RdRP (PDB ID: 6M71 [10]) using rigid docking. To further improve the binding scores of the rigid docking, flexible screenings were performed on the top ten hits by making the side chains flexible for residues Arg553, Arg555, Lys621, Asp623 and Ser814 in the RdRP tunnel region. Similarly, another round of flexible screenings was performed against the SARS-CoV-2 structure of RdRP using the top 10 hits from the ensemble docking. Vina from AutoDockTools 4.2 [29] was used to perform docking and screening. Top hits were sorted and ranked based on their binding energy scores using custom scripts.

## Results and discussion

Previously we identified potential antiviral targets in the key proteins in coronavirus such as MERS-CoV and SARS-CoV [7] that are also conserved in the novel coronavirus SARS-CoV-2. RdRP was found to have the greater amount of surface-accessible targets that were strategically located in the vicinity of the active site and RNA tunnel. RdRPs are established antiviral drug targets. To find drugs that could potentially inhibit RdRP in SARS-CoV-2, and consequently be used as a therapeutic for COVID-19, four different *in silico* screens of FDA-approved drugs were performed targeting the RNA tunnel.

### Phylogenetic analysis

**Fig. 1.**
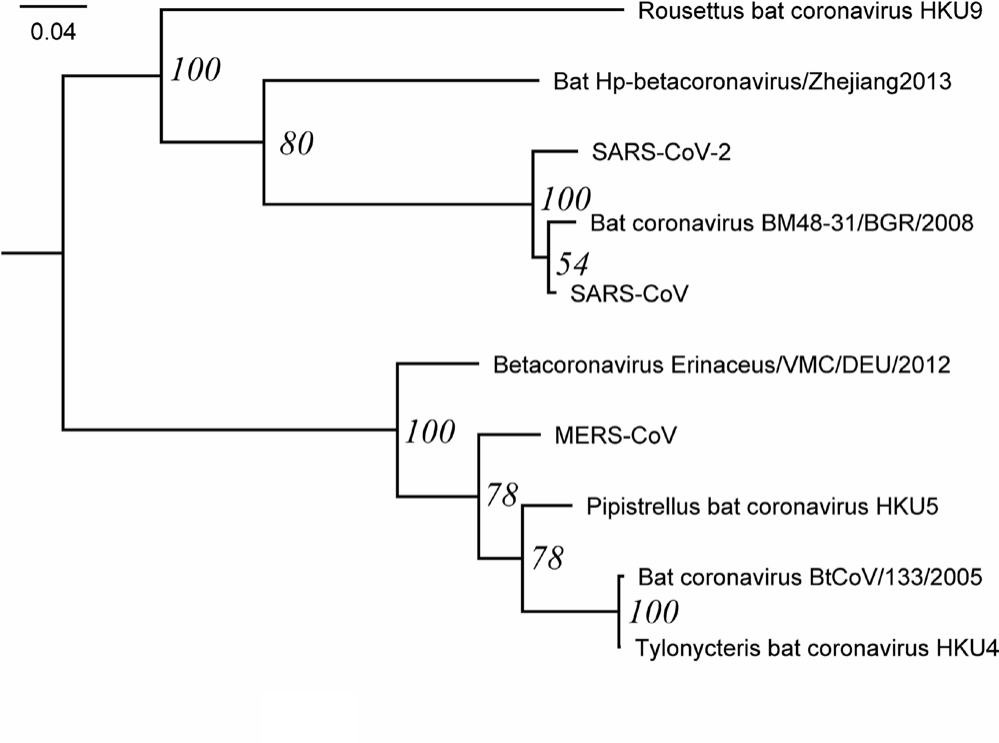
Phylogenetic tree of RdRP. The tree was inferred with PhyML using LG+G+I+F as the best model of evolution and 100 bootstraps, shown at the nodes. The tree was visualized and rooted at mid-point with FigTree.

The phylogeny of RdRP closely resembles the previous phylogeny [7], with SARS-CoV-2 as a sister to SARS-CoV and bat coronavirus-48, a SARS-related coronavirus isolated from a bat in Bulgaria in 2008 [32] (Fig. 1). The placement of SARS-CoV-2 is in agreement with the coronavirus phylogeny used to name the novel virus [3]. The phylogeny is overall well supported, but the clade formed by SARS-CoV and bat coronavirus-48 has low bootstrap support (54 %). Based on branch lengths, SARS-CoV-2 is more similar to SARS-CoV than to bat coronavirus-48.

### Expanded analysis of the targets in coronaviral proteins with an emphasis on RdRP

We found that all target motifs identified previously from other coronaviruses [7] were conserved in the SARS-CoV-2 reference sequence. In the comparison of all SARS-CoV-2 sequences, one amino acid substitution was found in one motif each for RdRP, helicase and exonuclease (Table 1). The overall conservation of the SARS-CoV-2 proteins for protease (NSP5), replicase (NSP7), RdRP (NSP12), helicase (NSP13) and exonuclease (NSP14) is close to 100 % (Fig. S1, available in the online version of this article).

**Table 1.** Antiviral targets

Protein	Target	Sequence motif* and SARS-CoV-2 numbering†	PDB ID‡, range
**Protease**	NSP5 : 1	3406-**GSC**GS-3387	1UJ1 : 143–147
**Protease**	NSP5 : 2	346-A**W**LYAA-3474	1UJ1 : 206–211
**Replicase**	NSP7 : 1	3866-**K**CTSWLL-3873	6NUR_C: 7–14
**Replicase**	NSP7 : 2	3875-VL**QQL**-3879	6NUR_C: 16–20
**RdRP**	NSP12 : 1	4599-**LDN**QDL**NG**-4606	6NUR_A: 207–214
**RdRP**	NSP12 : 2	4610-**DFGDF**-4614	6NUR_A: 218–222
**RdRP**	NSP12 : 3	4891-**DKS**AG-4895	6NUR_A: 499–503
**RdRP**	NSP12 : 4	4958-MTN**R**Q-4962	6NUR_A: 566–570
**RdRP**	NSP12 : 5	5047-LAN**EC** AQV**L**-5055	6NUR_A: 655–663
**RdRP**	NSP12 : 6	5070-GG**T**S**SGD**-5076	6NUR_A: 678–684
**RdRP**	NSP12 : 7	5223-**Y**P**DPSR**-5228	6NUR_A: 831–836
**RdRP**	NSP12 : 8	5241-**KTDGT**-5245	6NUR_A: 849–853
**RdRP**	NSP12 : 9	5259-**Y**PL**TK**-5263	6NUR_A: 867–871
**Helicase**	NSP13 : 1	5334-**SQ**T**S**L**R**-5339	6JYT: 10–15
**Helicase**	NSP13 : 2	5685-**NAL** **P** **E**-5689	6JYT: 361–365
**Helicase**	NSP13 : 3	5725-**DPAQLP**-5730	6JYT: 401–406
**Helicase**	NSP13 : 4	5859-**SSQGS**-5863	6JYT: 535–539
**Exonuclease**	NSP14 : 1	6192-**AHV**A**S**-6196	5C8S_B: 267–271
**Exonuclease**	NSP14 : 2	6201-MT**RCL**A-6206	5C8S_B: 276–280
**Exonuclease**	NSP14 : 3	6349-**H** **A** **FHT**-6353	5C8S_B: 424–428
**Exonuclease**	NSP14 : 4	6402-**CNLGG**-6406	5C8S_B: 477–481

*Sequence motifs in the targets provided by Rahaman and Siltberg-Liberles [7].

†Residues shown in bold are surface accessible. Residues that are not 100 % conserved across the SARS-CoV-2 strains are underlined.

‡PDB ID is given with chain if appropriate, e.g. 6NUR_A, means chain A from PDB ID 6NUR. References for PDB IDs: 1UJ1 [19], 6NUR [9], 6JYT [20] and 5C8S [21].

**Fig. 2.**
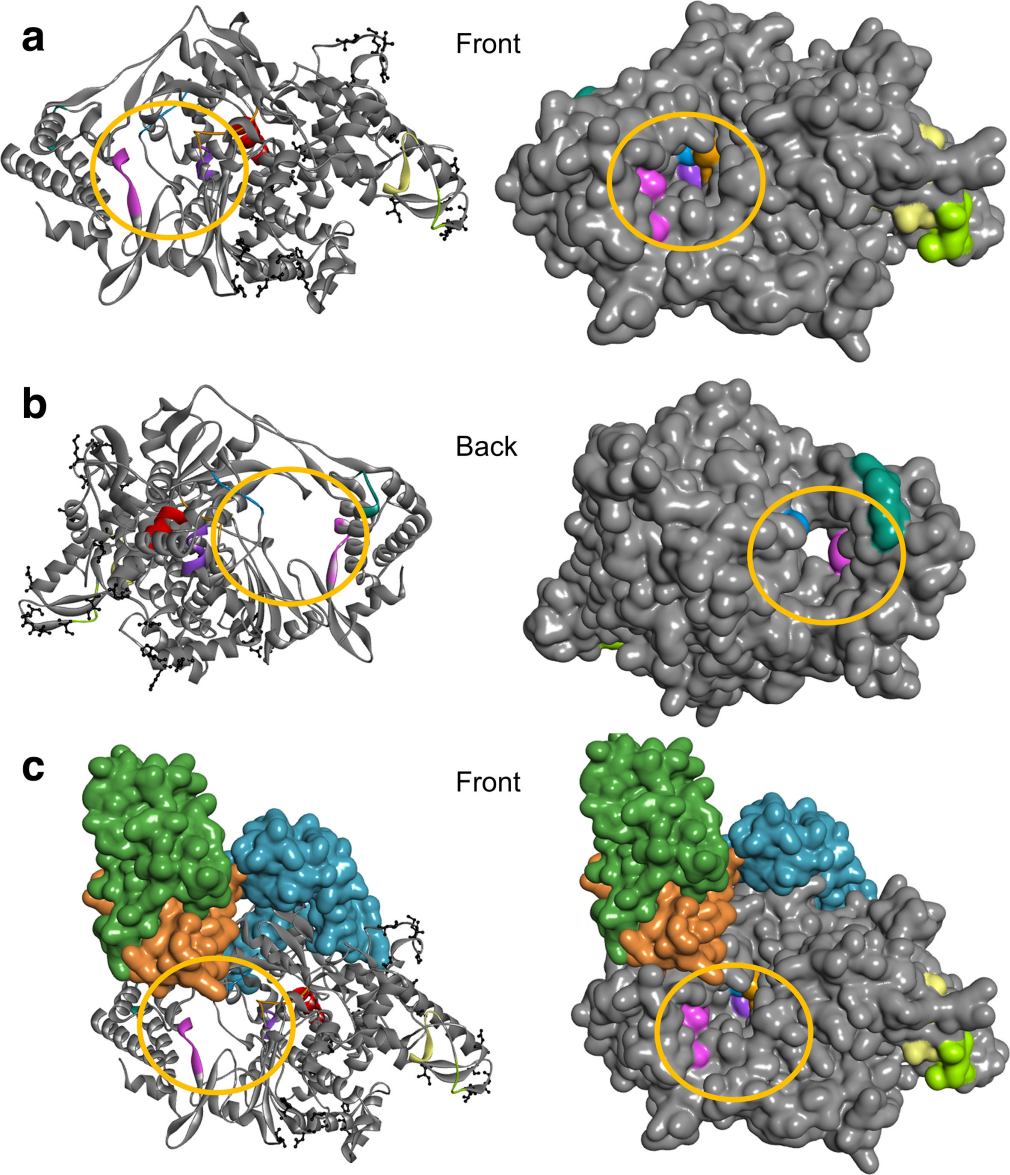
Sequence and structure comparison of SARS-CoV RdRP based on PDB ID 6NUR to SARS-CoV-2 RdRP (grey), shown as ribbon and as surface representation from the front (a), the back (b) and the front with its cofactors, NSP7 (orange) and NSP8 (two chains: blue, green) (c). Previously proposed target regions are colour coded (targets 1 to 9 are represented by yellow, green, blue, purple, red, orange, pink, teal and brown, respectively). The active site/tunnel (from here on referred to as the RNA tunnel: yellow circle) is open and ready to perform its function. The part of the RdRP sequence from SARS-CoV included in PDB ID 6NUR is 96 % identical to the corresponding sequence from SARS-CoV-2 according to blast. The differing residues are shown as ball and stick (black). Protein structure visualized by BIOVIA Discovery Studio Visualizer v. 16.1.

For a motif to be considered a drug target it must be surface-accessible. The number of residues that are surface-accessible for protease, replicase, RdRP, helicase and exonuclease is 4, 4, 34, 20 and 17, respectively (Table 1). RdRP was expected to have the most surface-accessible residues because it has nine different motifs, compared to two each for protease and replicase, and four each for helicase and exonuclease (Table 1). The targets for RdRP are clustered in and around the RNA tunnel (Fig. 2). Replicase (NSP7) acts as a cofactor for RdRP [9] and its targets are located at the interface between the two proteins (Fig. 2). The targets in helicase (NSP13) and exonuclease (NSP14) are surface accessible, whereas those in protease (NSP5) are mostly buried and appear poorly accessible to small molecules (Table 1). In summary, RdRP appears to be the ideal target with the highest number of surface-accessible and strategically placed drug-binding sites. Furthermore, RdRPs are emerging as antiviral targets for RNA viruses [33–35].

Sequence comparison of RdRP in SARS-CoV-2 to SARS-CoV (based on PDB ID: 6NUR) reveals that these are 96 % identical in amino acid sequence (Fig. S2) and structural analysis shows that the nonconserved amino acid residues are located away from the RNA tunnel that represents an essential element for its catalytic activity (Fig. 2). Importantly, the target regions, in the critical area around the RNA tunnel, are far from the nonconserved amino acids between SARS-CoV and SARS-CoV-2 (Fig. 2).

### Virtual screening of RdRP for drug binding

Ensembled-based molecular docking was performed for the 1930 FDA-approved drugs [30] against 200 conformations of SARS-CoV RdRP (based on PDB ID: 6NUR [9]). The top-scoring drugs generally had good binding scores on multiple protein conformations. A ranked list is prepared for distinct drugs with their best scores for the drug–protein conformations. Rigid molecular docking was performed for the 1930 FDA-approved drugs [30] against 1 conformation of SARS-CoV-2 RdRP (PDB ID: 6M71 [10]). Flexible docking, where the side chains of Lys551, Arg553, Arg555, Lys621, Asp623 and Ser814 in the RNA tunnel of RdRP in SARS-CoV-2 were kept flexible, was performed for the top 10 hits from the rigid docking and from the ensemble docking, respectively. Several drugs are identified in all the different docking rounds (Table 2).

**Table 2.** Top-scoring drugs from the different docking rounds*

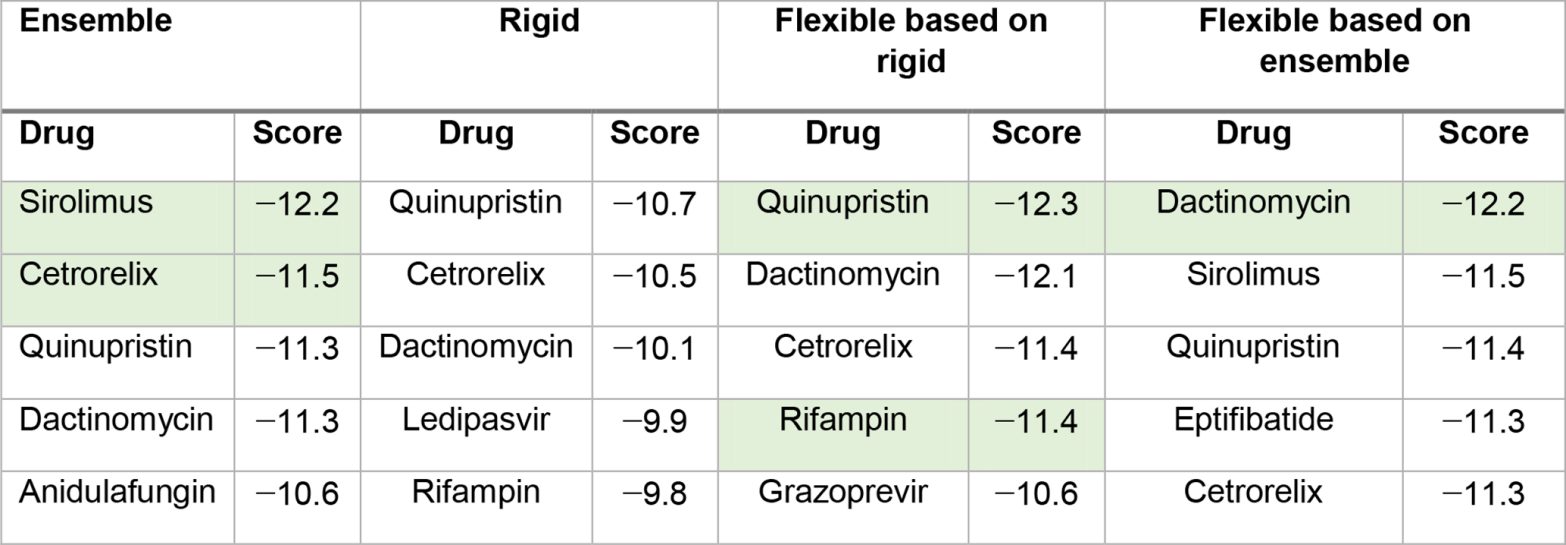

*The lowest (best) score for the five top-ranked drugs are shown in green.

**Fig. 3.**
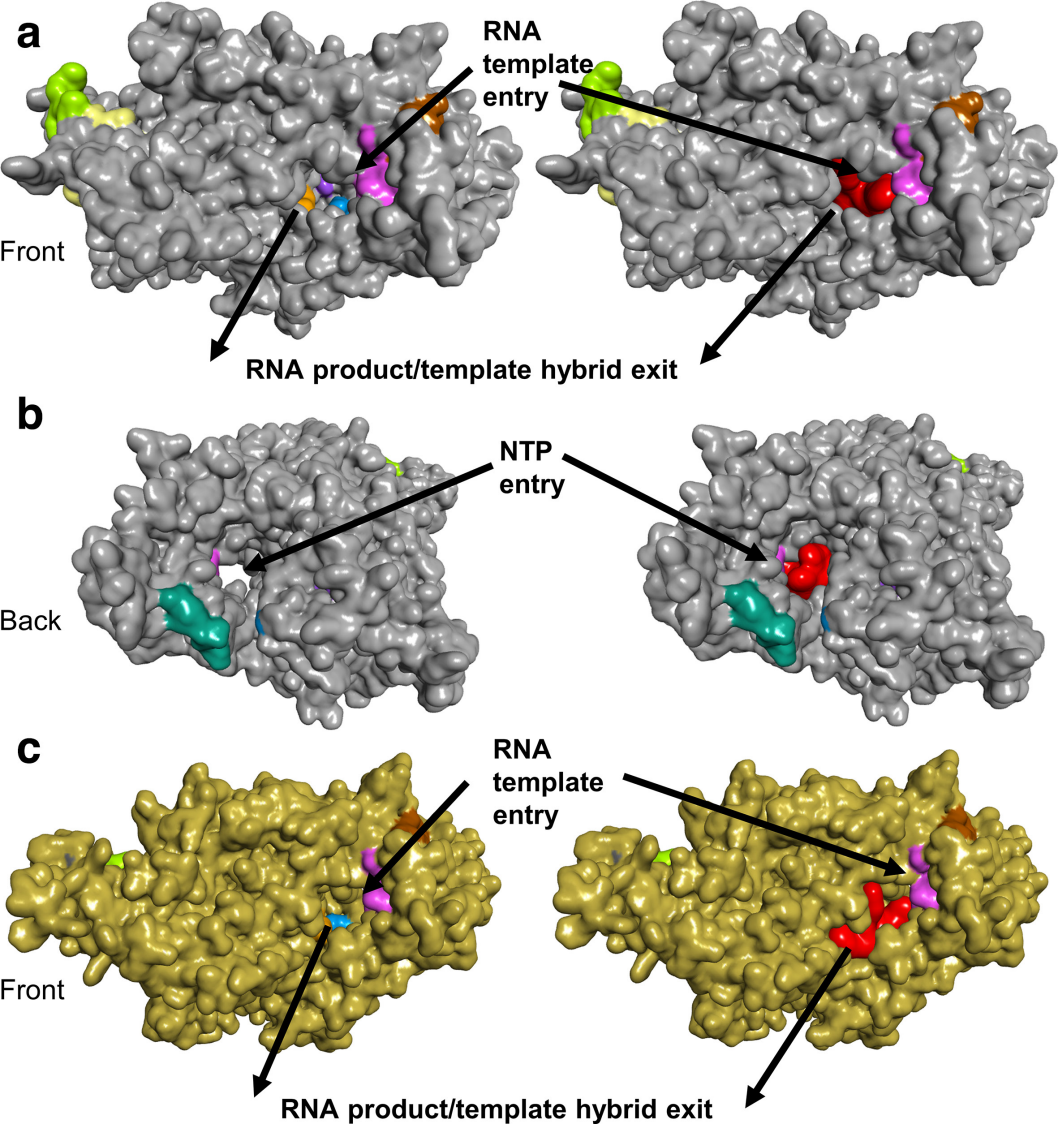
Surface representations of RdRP with quinupristin. The top-ranked RdRP SARS-CoV (grey) conformation from the ensemble docking (PDB ID: 6NUR) is shown with (right) and without (left) quinupristin (red) from the front (a) and the back (b). The top-ranked RdRP SARS-CoV-2 (gold) from the flexible docking round (PDB ID: 6M71) with (right) and without (left) quinupristin (red) from the front (c). Quinupristin is buried in the RNA tunnel, blocking access for NTP and RNA. Previously proposed target regions are colour coded (targets 1 to 9 are represented by yellow, green, blue, purple, red (buried, not shown), orange, pink, teal and brown, respectively). Protein structure and drug complexes visualized by BIOVIA Discovery Studio Visualizer v. 16.1.

**Fig. 4.**
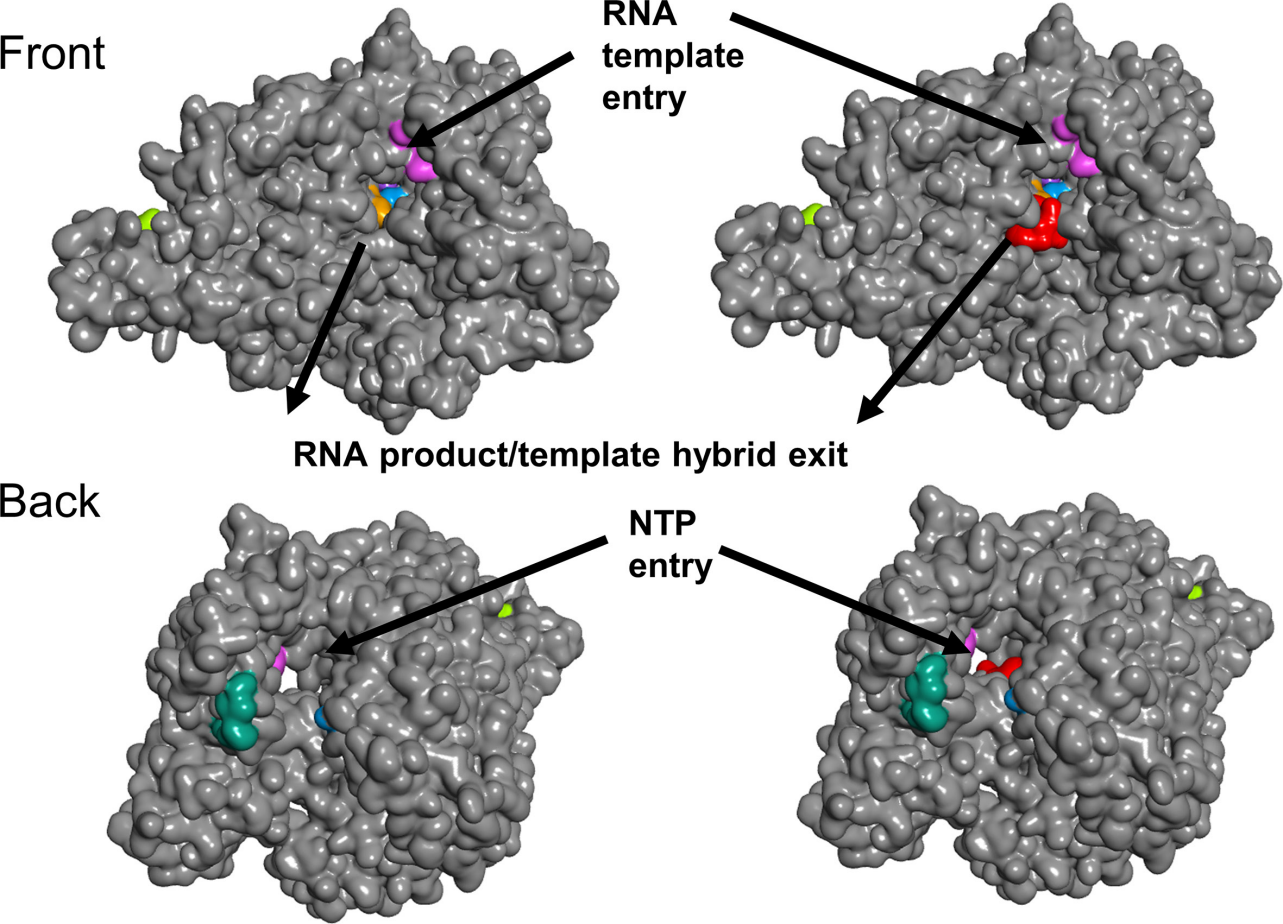
Surface representations of RdRP with rifampin. The top-ranked RdRP SARS-CoV-2 (grey) conformation from the flexible docking (PDB ID: 6M71) is shown with (right) and without (left) rifampin (red) from the front (a) and the back (b). Previously proposed target regions are colour coded (targets 1 to 9 are represented by yellow, green, blue, purple, red (buried, not shown), orange, pink, teal and brown, respectively). Protein structure and drug complexes visualized by BIOVIA Discovery Studio Visualizer v. 16.1.

The top-ranked candidate based on the virtual screens is the antibiotic quinupristin (Table 3). The *in silico* screens suggest that quinupristin binds across the RNA tunnel (Fig. 3), potentially blocking access for nucleoside triphosphates (NTPs) to enter the active site on one side and for RNA on the other. Without NTPs, RNA replication cannot occur. If the RdRP function can be inhibited, this may block viral replication. The second and third candidates, dactinomycin and sirolimus (rapamycin), both block the RNA tunnel, but have cytotoxic and immunosuppressive properties, respectively, preventing their use as antimicrobials [36, 37]. The fourth candidate, cetrorelix, inhibits the effects of gonadotropin-releasing hormone, is used during fertility treatments, and may have undesirable side effects for some populations. Cetrorelix also binds across the RNA tunnel. The fifth candidate, rifampin, is a broad-spectrum antibiotic that is often used to treat mycobacterial infections such as tuberculosis [37]. Our *in silico* screen places rifampin on the side of the RNA tunnel, in the vicinity of the RNA product/template hybrid exit (Fig. 4).

**Table 3.** Top five hits from virtual screening with FDA-approved drugs against RdRP

Final rank and Best score*	Drug name	DrugBank† description
1	Quinupristin	An antibiotic effective against Gram-positive bacteria; commonly used in combination with dalfopristin
Score: −12.3		
2	Dactinomycin	An antibiotic that inhibits bacterial transcription; has cytotoxic properties; used for chemotherapy
Score: −12.2		
3	Sirolimus	A macrolide from *Streptomyces hygroscopicus* with immunosuppressive, antifungal and antineoplastic properties
Score: −12.2		
4	Cetrorelix	A man-made hormone that blocks the effects of gonadotropin-releasing hormone
Score: −11.5		
5	Rifampin	A broad-spectrum antibiotic known to inhibit DNA-dependent RNA polymerase activity by forming a stable complex with the enzyme
Score: −11.4		

*Best score based on the virtual screens (Table 2).

†DrugBank [36, 37].

**Fig. 5.**
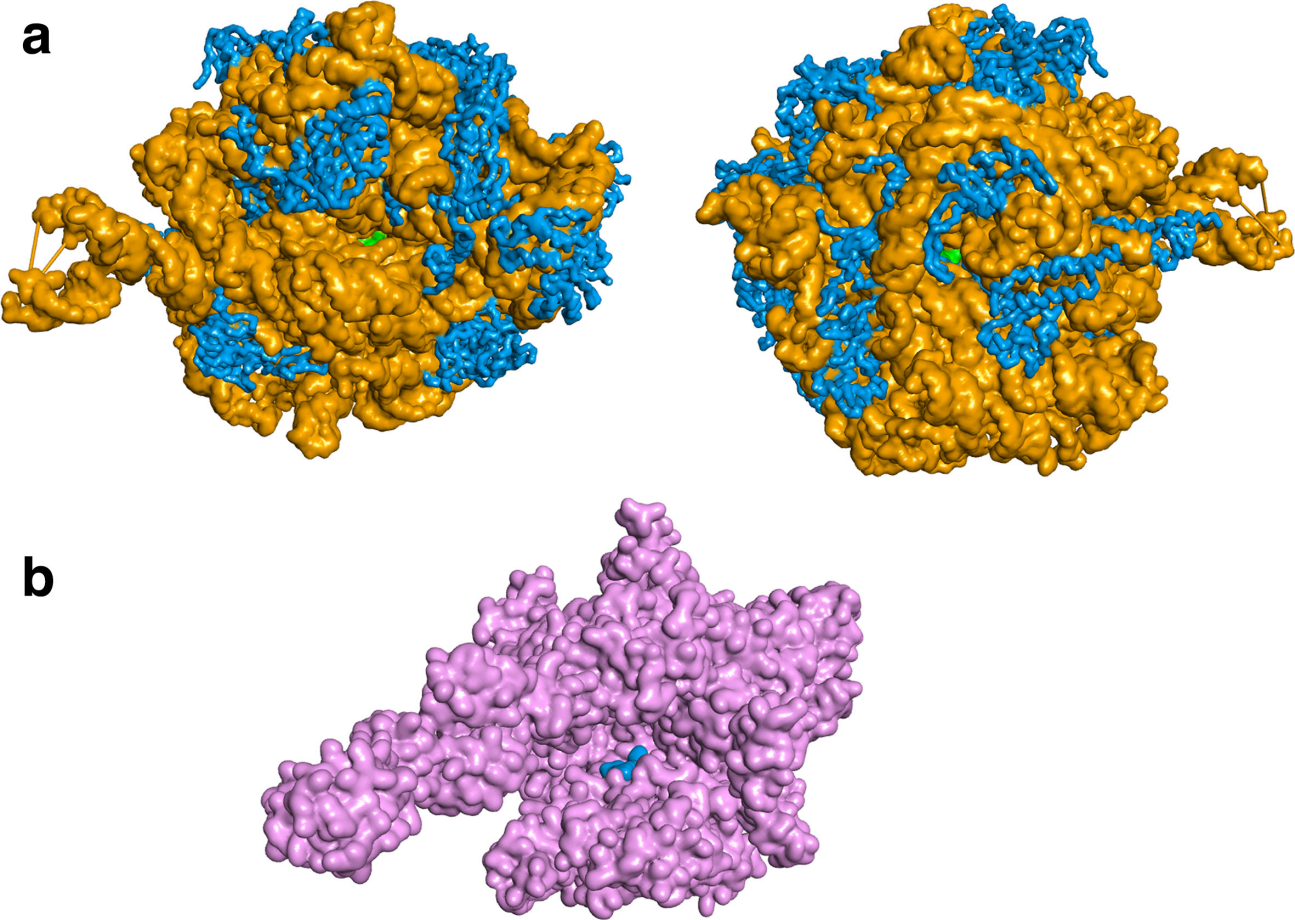
Quinupristin and rifampin bound to their intended drug targets based on co-crystalized X-ray structures. Quinupristin (green) has been suggested to inhibit the bacterial 50S ribosomal subunit by blocking the exit channel (a) (PDB ID: 1sm1 [39]). The bacterial 50S is a ribozyme and consists of protein (blue) and RNA (orange). Rifampin (blue) binds to one side of the channel leading up to the active site of the bacterial RNA polymerase (pink), blocking the path of elongation (b) (PDB ID: 1ynn [52]). Protein structure and drug complexes visualized by BIOVIA Discovery Studio Visualizer v. 16.1.

Our five best-ranked candidates include two antibiotics that directly target RNA-binding and ribosomal regions in bacterial proteins such as the DNA-dependent RNA polymerase (rifampin) [38] or riboproteins such as the 50S unit of the ribosome (quinupristin) (Fig. 5) [39]. While the RNA polymerase and the ribosome are two diverse biomolecules, two common denominators are RNA and functionality that depend on a tunnel, two traits shared with the coronavirus RdRP. Rifampin and quinupristin prevent bacterial gene expression and protein translation, respectively. These antibiotics were not designed to act on the human host. Rifampin’s mechanism of action is to inhibit bacterial DNA-dependent RNA polymerase by binding to the side of the DNA/RNA channel that leads to the active site [38]. Rifampin binds away from the active site of the bacterial RNA polymerase in an area that is not conserved in eukaryotes [40]. Rifampin has good bioavailability and low toxicity, but is frequently used in combination with other antimicrobials to avoid antibiotic resistance [41]. Quinupristin binds to the prokaryotic 50S subunit of the ribosome, which is smaller than the eukaryotic 60S subunit and only partially homologous [42]. Quinupristin, together with dalfopristin, is part of the two-antibiotic mixture Synercid that has proven to be as safe in adults ≥65 years of age as in younger adults [43], which is important because COVID-19 has posed a particularly high risk of mortality to the elderly. Quinupristin and dalfopristin are streptogramin antibiotics known to act in concert to inhibit bacterial protein synthesis. Quinupristin and dalfopristin have been found to bind in, and block, the ribosomal exit tunnel in the 50S bacterial ribosome [39].

Currently, Synercid is used against methicillin-resistant forms of *Staphylococcus aureus* (MRSA) and other severe bacterial infections [44]. As an antibiotic, the synergistic effect of quinupristin and dalfopristin is needed for it to be effective. Combinations of Synercid with rifampin have been found to have an even stronger synergistic effect against MRSA and macrolide–lincosamide–streptogramin B-resistant (MLSB) isolates [45]. It is possible that a combination of quinupristin (or Synercid) and rifampin are more effective together as an antiviral against COVID-19 because, based on the *in silico* results, they may bind to different regions of the RNA tunnel in the coronavirus RdRP (Figs 3 and 5). It must be noted that we do not know if quinupristin or rifampin will bind to RdRP *in vitro* or *in vivo*, and the efficacy of either one or both in combination is yet to be demonstrated.

## Conclusion

Molecular docking predictions have become increasingly useful, not only due to the accuracy in energy functions as well as advancement in computational speed, but also because of recent developments in cryo-EM technology. The structure of SARS-CoV RdRP that we used was the first coronavirus RdRP structure and it was released last year [9]. Currently, we have two similar RdRP structures, one for SARS-CoV and one for SARS-CoV-2 [10]. Without structural representatives, molecular docking is not possible. Molecular docking is frequently used for *in silico* screens of many drugs against a protein target as part of the initial steps of drug discovery [46]. Drug repurposing is a subfield of drug discovery that is focused on the identification of known drugs that can potentially bind to new targets. Drug repurposing often means lower risk of failure and a shorter preclinical phase [47]. Virtual, *in silico*, predictions are increasingly being used to guide the initial selections of compounds for *in vitro* analysis. Given the limited number of compounds in the database, it is possible that none of the drugs in the database will bind to our target *in vivo*. However, if we are to look for a best-binding compound in the database of FDA-approved drugs, these predictions are reasonably good. We used three types of docking (ensemble, rigid and flexible) to perform *in silico* screens. Ensemble docking better describes the nature of a protein’s structure by considering a conformational ensemble, but it is computationally expensive [48]. Rigid docking considers only one static conformation of the protein, but it is computationally attractive. Flexible docking allows for some residues explore their conformational space during the docking and can be used to improve binding scores. Notably, all three types returned quinupristin as a top-ranked candidate.

The *in silico* predicted drug candidates presented here can be considered for *in vitro* and *in vivo* tests and we hope that further exploration of such compounds and their analogues will allow researchers to quickly identify much-needed antiviral therapeutics for COVID-19. It is important to note that other coronaviruses that are yet unknown to humans could still pose future threats [46]. Under a different scenario, the potential broadly neutralizing antiviral target regions (Table 1) can be further pursued by a wider range of drug-like compounds in other libraries for future long-term drug development purposes. By utilizing data (genomic, structural, medical, etc.), viral precision medicine can be utilized to avoid playing catch-up with the next emerging coronavirus.

Given the urgency of the COVID-19 pandemic, we focused this screen on the library of already clinically used FDA-approved drugs aiming for drug repurposing. We chose to focus on RdRP because it is a known antiviral target for other RNA viruses such as hepatitis C virus [35]. Since this study began, it has been shown that MERS-CoV replication can be controlled by remdesivir in rhesus monkeys [5]. Remdesivir is an RdRP inhibitor that was developed for Ebola [49]. Remdesivir was not shown to be effective against Ebola, but it is currently included in the SOLIDARITY clinical trial for coronavirus treatments that the World Health Organization (WHO) recently announced [50]. If quinupristin, and perhaps rifampin, are indeed able to inhibit RdRP, these are RdRP inhibitors that could be evaluated rapidly as therapeutics for COVID-19. Lastly, a recent study found that 50 % of hospitalized COVID-19 patients who did not survive had secondary infections [51]. Perhaps these antibiotic candidates will have benefits that go beyond the COVID-19 infection itself.

## Supplementary Data

Supplementary material 1
