# Supplementary material for: Potential RNA-dependent RNA polymerase inhibitors as prospective therapeutics against SARS-CoV-2
Source: J Med Microbiol. 2020 May 29;69(6):864–73. doi: 10.1099/jmm.0.001203 (PMC7451031; doi:10.1099/jmm.0.001203)
Supplement: Supplementary material 1 [file jmm-69-864-s001.pdf]

Supplementary material

Fig S1. SARS-CoV-2 strain comparisons. Heatmaps showing pairwise sequence of SARS-CoV-2 sequences from GenBank.

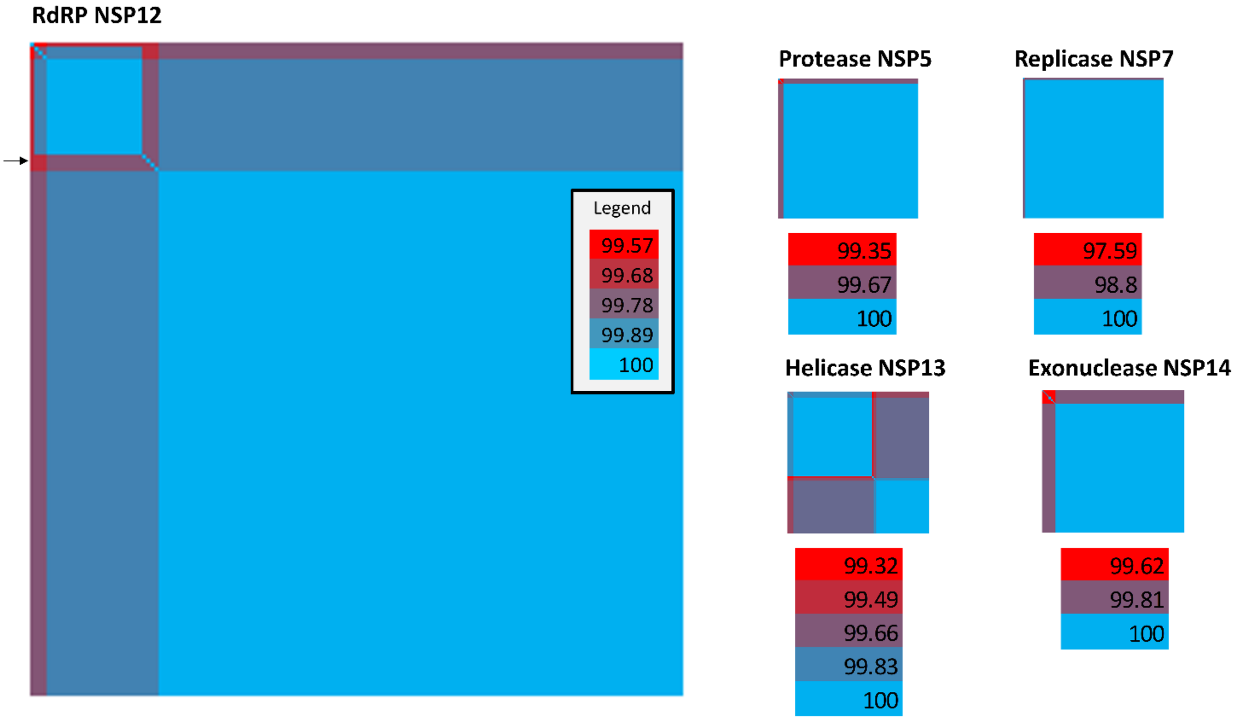

As of 4/09/2020, GenBank had 164 sequences for the RdRP from SARS-CoV-2 with at least 95% query cover to the RdRP region of the reference sequence from SARS-CoV-2 (YP_009724389.1, range 4398-5324). Sequences with X characters were excluded from the analysis.

The heatmaps above show a comparison of percent sequence identity for the remaining 157 RdRP sequences based on the symmetrical percent identity matrix from a multiple sequence alignment by Muscle [1] from EBI [2]. The color code is shown in the insert.

The target motifs are conserved with one exception in target motif 5 where there is an A to S substitution in the 5th position of the motif. The sequence with this substitution (accession number BCB97890.1) was isolated in Japan and its location in the heatmap is marked by the black arrow.

Similarly, for NSP5, 211 sequences without X were available in GenBank. Both targets were 100% conserved.

NSP7, 214 sequences without X were available in GenBank. Both targets were 100% conserved.

NSP13, 162 sequences without X were available in GenBank. Three targets were 100% conserved. Two sequences had a substitution in the 4th position of target 2 (P to S).

NSP14, 164 sequences without X were available in GenBank. Three targets were 100% conserved but target 3 had an amino acid substitution in the 2nd position (A to T) in one sequence.

Fig. S2. Sequence comparison of SARS-CoV based on PDB id 6NUR [3] to SARS-CoV-2. The part of NSP12 sequence from SARS-CoV included in PDB id 6NUR is 96% identical to the corresponding sequence from SARS-CoV-2 according to BLAST. The target regions are colored by motif.

BLAST alignment:

Query: 6NUR (seqres portion)
Subject: orf1ab polyprotein [Severe acute respiratory syndrome coronavirus 2]
Sequence ID: YP_009724389.1 Length: 7096
Range 1: 4509 to 5311

Alignment statistics for match #1

Score	Expect	Method	Identities	Positives	Gaps
1625 bits(4207)	0.0	Compositional matrix adjust.	769/803(96%)	781/803(97%)	10/803(1%)

```
Query  1     QRLTKYTMADLVYALRHFDEGNCDTLKEILVTYNCCDDDYFNKKDWYDFVENPDILRVYA  60
             QRLTKYTMADLVYALRHFDEGNCDTLKEILVTYNCCDDDYFNKKDWYDFVENPDILRVYA
Sbjct  4509  QRLTKYTMADLVYALRHFDEGNCDTLKEILVTYNCCDDDYFNKKDWYDFVENPDILRVYA  4568

Query  61    NLGERVRQSLLKTVQFCDAMRDAGIVGVLTLDNQDLNGNWYDFGDFVQVAPGCGVPIVDS  120
             NLGERVRQ+LLKTVQFCDAMR+AGIVGVLTLDNQDLNGNWYDFGDF+Q  PG GVP+VDS
Sbjct  4569  NLGERVRQALLKTVQFCDAMRNAGIVGVLTLDNQDLNGNWYDFGDFIQTTPGSGVPVVDS  4628

Query  121   YYSLLMPILTLTRALAAESHMDADLAKPLIKWDLLKYDFTEERLCLFDRYFKYWDQTYHP  180
             YYSLLMPILTLTRAL AESH+D DL KP IKWDLLKYDFTEERL LFDRYFKYWDQTYHP
Sbjct  4629  YYSLLMPILTLTRALTAESHVDTDLTKPYIKWDLLKYDFTEERLKLFDRYFKYWDQTYHP  4688

Query  181   NCINCLDDRCILHCANFNVLFSTVFPPTSFGPLVRKIFVDGVPFVVSTGYHFRELGVVHN  240
             NC+NCLDDRCILHCANFNVLFSTVFPPTSFGPLVRKIFVDGVPFVVSTGYHFRELGVVHN
Sbjct  4689  NCVNCLDDRCILHCANFNVLFSTVFPPTSFGPLVRKIFVDGVPFVVSTGYHFRELGVVHN  4748

Query  241   QDVNLHSSRLSFKELLVYAADPAMHAASGNLLLDKRTTCFSVAALTNNVAFQTVKPGNFN  300
             QDVNLHSSRLSFKELLVYAADPAMHAASGNLLLDKRTTCFSVAALTNNVAFQTVKPGNFN
Sbjct  4749  QDVNLHSSRLSFKELLVYAADPAMHAASGNLLLDKRTTCFSVAALTNNVAFQTVKPGNFN  4808

Query  301   KDFYDFAVSKGFFKEGSSVELKHFFFAQDGNAAISDYDYYRYNLPTMCDIRQLLFVVEVV  360
             KDFYDFAVSKGFFKEGSSVELKHFFFAQDGNAAISDYDYYRYNLPTMCDIRQLLFVVEVV
Sbjct  4809  KDFYDFAVSKGFFKEGSSVELKHFFFAQDGNAAISDYDYYRYNLPTMCDIRQLLFVVEVV  4868

Query  361   DKYFDCYDGGCINANQVIVNNLDKSAGFPFNKWGKARLYYDSMSYEDQDALFAYTKRNVI  420
             DKYFDCYDGGCINANQVIVNNLDKSAGFPFNKWGKARLYYDSMSYEDQDALFAYTKRNVI
Sbjct  4869  DKYFDCYDGGCINANQVIVNNLDKSAGFPFNKWGKARLYYDSMSYEDQDALFAYTKRNVI  4928

Query  421   PTITQMNLKYAISAKNRARTVAGVSICSTMTNRQFHQKLLKSIAATRGATVVIGTSKFYG  480
             PTITQMNLKYAISAKNRARTVAGVSICSTMTNRQFHQKLLKSIAATRGATVVIGTSKFYG
Sbjct  4929  PTITQMNLKYAISAKNRARTVAGVSICSTMTNRQFHQKLLKSIAATRGATVVIGTSKFYG  4988

Query  481   GWHNMLKTVYSDVETPHLMGWDYPKCDRAMPNMLRIMASLVLARKHNTCCNLSHRFYRLA  540
             GWHNMLKTVYSDVE PHLMGWDYPKCDRAMPNMLRIMASLVLARKH TCC+LSHRFYRLA
Sbjct  4989  GWHNMLKTVYSDVENPHLMGWDYPKCDRAMPNMLRIMASLVLARKHTTCCSLSHRFYRLA  5048

Query  541   NECAQVLSEMVMCGGSLYVKPGGTSSGDATTAYANSVFNICQAVTANVNALLSTDGNKIA  600
             NECAQVLSEMVMCGGSLYVKPGGTSSGDATTAYANSVFNICQAVTANVNALLSTDGNKIA
Sbjct  5049  NECAQVLSEMVMCGGSLYVKPGGTSSGDATTAYANSVFNICQAVTANVNALLSTDGNKIA  5108

Query  601   DKYVRNLQHRLYECLYRNRDVDHEFVDEFYAYLRKHFSMMILSDDAVVCYNSNYAAQGLV  660
             DKYVRNLQHRLYECLYRNRDVD +FV+EFYAYLRKHFSMMILSDDAVVC+NS YA+QGLV
Sbjct  5109  DKYVRNLQHRLYECLYRNRDVDTDFVNEFYAYLRKHFSMMILSDDAVVCFNSTYASQGLV  5168

Query  661   ASIKNFKAVLYYQNNVFMSEAKCWTETDLTKGPHEFCSQHTMLVKQGDDYVYLPYPDPSR  720
             ASIKNFK+VLYYQNNVFMSEAKCWTETDLTKGPHEFCSQHTMLVKQGDDYVYLPYPDPSR
Sbjct  5169  ASIKNFKSVLYYQNNVFMSEAKCWTETDLTKGPHEFCSQHTMLVKQGDDYVYLPYPDPSR  5228

Query  721   ILGAGCFVDDIVKTDGTLMIERFVSLAIDAYPLTKHPNQEYADVFHLYLQYIRKLHDEL-  779
             ILGAGCFVDDIVKTDGTLMIERFVSLAIDAYPLTKHPNQEYADVFHLYLQYIRKLHDEL
Sbjct  5229  ILGAGCFVDDIVKTDGTLMIERFVSLAIDAYPLTKHPNQEYADVFHLYLQYIRKLHDELT  5288

Query  780   ---------MLTNDNTSRYWEPE  793
                      MLTNDNTSRYWEPE
Sbjct  5289  GHMLDMYSVMLTNDNTSRYWEPE  5311
```

References

1. **Edgar RC**. MUSCLE: a multiple sequence alignment method with reduced time and space complexity. *BMC Bioinformatics* 2004;5:113.

2. **Madeira F, Park YM, Lee J, Buso N, Gur T, *et al.*** The EMBL-EBI search and sequence analysis tools APIs in 2019. *Nucleic Acids Res*. Epub ahead of print 12 April 2019. DOI: 10.1093/nar/gkz268.

3. **Kirchdoerfer RN, Ward AB**. Structure of the SARS-CoV nsp12 polymerase bound to nsp7 and nsp8 co-factors. *Nat Commun*;10. Epub ahead of print 1 December 2019. DOI: 10.1038/s41467-019-10280-3.
